# Morpho-molecular characterization of *Discosia ravennica* sp. nov. and a new host record for *Sporocadus rosigena*

**DOI:** 10.3897/mycokeys.79.60662

**Published:** 2021-04-27

**Authors:** Digvijayini Bundhun, Rajesh Jeewon, Indunil C. Senanayake, Erio Camporesi, Janith V. S. Aluthmuhandiram, Alvin M. C. Tang, Ji-Chuan Kang, Vishwakalyan Bhoyroo, Kevin D. Hyde

**Affiliations:** 1 Engineering and Research Center for Southwest Bio-Pharmaceutical Resources of National Education Ministry of China, Guizhou University, Guiyang, Guizhou Province 550025, China; 2 Center of Excellence in Fungal Research, Mae Fah Luang University, Chiang Rai, 57100, Thailand; 3 Department of Entomology and Plant Pathology, Faculty of Agriculture, Chiang Mai University, Chiang Mai, 50200, Thailand; 4 Department of Health Sciences, Faculty of Medicine and Health Sciences, University of Mauritius, Reduit, Mauritius; 5 College of Life Science and Oceanography, Shenzhen University, 1068, Nanhai Avenue, Nanshan, Shenzhen, 518055, China; 6 Innovative Institute of Plant Health, Zhongkai University of Agriculture and Engineering, Haizhu District, Guangzhou, 510225, China; 7 A.M.B. Gruppo Micologico Forlivese “Antonio Cicognani”, Via Roma, Forli, Italy; 8 A.M.B. Circolo Micologico “Giovanni Carini”, Brescia, Italy; 9 Società per gli Studi Naturalistici della Romagna, Bagnacavallo (RA), Italy; 10 Beijing Key Laboratory of Environment Friendly Management on Fruit Diseases and Pests in North China, Institute of Plant and Environment Protection, Beijing Academy of Agriculture and Forestry Sciences, Beijing, 100097, China; 11 Division of Applied Science, College of International Education, Hong Kong Baptist University, Hong Kong SAR, China; 12 Faculty of Agriculture, University of Mauritius, Reduit, Mauritius

**Keywords:** Amphisphaeriales, asexual morphs, new species, saprobes, taxonomy

## Abstract

Collections of fungal samples from two dead leaf specimens from Italy were subjected to morphological examination and phylogenetic analyses. Two coelomycetous taxa belonging to two different genera in Xylariomycetidae, Sordariomycetes, namely *Discosia* and *Sporocadus*, were identified. The *Discosia* taxon is revealed as a new species and is herein introduced as *Discosia ravennica***sp. nov.** while the *Sporocadus* taxon is identified as *Sporocadus rosigena*. Multi-locus phylogeny based on DNA sequence data of the large subunit (LSU) and internal transcribed spacer (ITS) of nuclear ribosomal genes, β-tubulin (*β-tub*) and RNA polymerase II second largest subunit (*rpb2*) showed that *D. ravennica* is related to *D. neofraxinea* but it forms an independent lineage that supports its new species status. The new taxon also differs from other *Discosia* species by its unilocular to bilocular, superficial and applanate conidiomata with basal stroma composed of cells of *textura angularis*, elongate-ampulliform conidiogenous cells and conidia smaller in size. *Sporocadus rosigena* is here reported as a new host record from *Quercus ilex* from Italy. Descriptions, illustrations and molecular data for both species are provided in this paper.

## Introduction

Members of the Sporocadaceae (Amphisphaeriales, Sordariomycetes) are generally appendage-bearing coelomycetes equally known as “pestalotioid fungi” (Tanaka et al. 2011; Liu et al. 2019). *Discosia* Lib. ex Durieu & Mont. and *Sporocadus* Corda are two genera in this family and they were shown to be phylogenetically linked as sister taxa (Jeewon et al. 2002; Maharachchikumbura et al. 2016).

After Libert (1837) established *Discosia*, it was re-studied by Subramanian and Reddy (1974) who designated *D. strobilina* Lib. ex Sacc. as lectotype for the genus (Nag Raj 1993; Tanaka et al. 2011). Later, when *Sphaeria artocreas* Tode was transferred to the genus and combined under *D. artocreas* (Tode) Fr., the latter was chosen as lectotype of the genus (Fries 1849; Vanev 1991). Morgan-Jones (1964) investigated both *D. artocreas* [same material examined by Fries (1849)] and *D. strobilina* and reported them as two different species. Subramanian and Reddy (1974) did not examine the type of *D. artocreas*, but the features of *D. strobilina* they observed did not match the same reported by Morgan-Jones (1964). The status of *D. artocreas* as type species of *Discosia*, therefore, has not been confirmed (Sutton 1980). Nevertheless, it is currently accepted as the type species of the genus (Crous et al. 2013; Index Fungorum, http://www.indexfungorum.org/Names/Names.asp). Recently, an epitype for *D. artocreas* was designated (Liu et al. 2019).

Delineation of *Discosia* taxa was earlier, primarily focused on morphological characteristics such as septation of the conidia, varying proportional lengths of the conidial cells and the conidium size (Subramanian and Reddy 1974; Sutton 1980; Vanev 1991, 1992, 1996; Nag Raj 1993). However, these similar morphological characters have been found to be overlapping for most *Discosia* species (Sutton 1977, 1980; Nag Raj 1993; Jeewon et al. 2002; Barber et al. 2011; Tanaka et al. 2011). Species of *Discosia* were earlier also divided into four sections based on the size, septation and pigmentation of the conidia (Subramanian and Reddy 1974). Later, six sections for the species were proposed based on the same conidial morphology (Vanev 1991). Acquisition of DNA sequence data for *Discosia* species followed by phylogenetic analyses have, however, shown that the concept of subdivision based on morphology alone has been inaccurate and that proper delineation of species must rely on both morphology and molecular phylogeny (Tanaka et al. 2011).

*Sporocadus* is a recently resurrected genus, characterized by integrated or discrete conidiogenous cells and generally 3-septate, ellipsoid, cylindrical or obovoid conidia which lack appendages (Liu et al. 2019). The genus was originally introduced to accommodate four species, including *S. herbarum* Corda, *S. georginae* Corda, *S. lichenicola* Corda and *S. maculans* Corda (Corda 1839). No type species for the genus was designated when these species were introduced. However, *S. lichenicola* was chosen as the lectotype by Hughes (1958). Although Wijayawardene et al. (2016) followed the synonymy of *Sporocadus* under *Seimatosporium* by Sutton (1975), Brockman (1976) and Nag Raj (1993) did not accept this. Recently, multi-loci phylogenetic analyses showed that *Sporocadus* and *Seimatosporium* are two separate genera, with the former genus usually accommodating taxa without appendages and epitypified by *S. lichenicola* (Liu et al. 2019).

Documenting fungal species, whether they are novel species or new records, is an important contribution to diversity, taxonomy and plant pathology. It is also imperative that these fungal taxa are studied as a number of them are recognized to be potential emerging plant pathogens and they can impact on disease management strategies (Dugan et al. 2009; Giraud et al. 2010; Ghelardini et al. 2016; Rodeva et al. 2016; Jayasiri et al. 2019; Jayawardena et al. 2020). The aim of this paper is to introduce a new *Discosia* species collected from Italy based on morphology supported by phylogenetic analyses of combined LSU, ITS, *β-tub* and *rpb2* sequence data. In addition, we report a new host record for a sporocadus-like taxon, identified as *Sporocadus rosigena*, isolated from *Quercus ilex* (Fagaceae) in Italy.

## Materials and methods

### Sample collection and isolation

Samples of plant materials bearing discosia-like and sporocadus-like fungi were collected from dead land leaves of *Pyrus* sp. and *Quercus ilex* in the provinces of Ravenna, Oriolo dei Fichi– Faenza and Forlì-Cesena, Fiumana di Predappio, Italy, respectively. They were brought to the laboratory in paper bags and labelled initially as IT 3632 and IT 3569. The specimens were then examined using a dissecting microscope (Motic SMZ-168).

Single-spore isolation was carried out as described in Senanayake et al. (2020). Conidia of the sporocadus-like taxon successfully germinated and were transferred aseptically to malt extract agar (MEA) plates. The cultures were incubated at 18 °C for 2–3 weeks with frequent observations to assess the colony color and other characters.

### Morphological studies

Free-hand sections of conidiomata of the *Discosia* taxon were prepared to examine their morphological characters. The following structures were observed and measured: height, diameter, and shape of conidiomata, conidiomatal wall cell structure, shape and dimensions of conidiophores and conidiogenous cells, length and width of conidia. Morphology of the representatives of the *Sporocadus* species was obtained from the culture and the morphological characters examined included conidiomata, conidiophores, conidiogenous cells and conidia. All the fungal characters were examined with a fluorescence microscope (Nikon Eclipse E600) and digital images were captured with a Nikon DS-U2 and Cannon 750D camera. All measurements were made using the Tarosoft (R) Image Frame Work software v.0.9.0.7. Images used for photo plates were processed with Adobe Photoshop CS6 v. 12.0 (Adobe Systems, USA).

### Material deposition

The holotype of the newly described taxon herein was deposited in the Mae Fah Luang University Herbarium (MFLU), Chiang Rai, Thailand while the isotype at the Cryptogamic Herbarium, Kunming Institute of Botany Academia Sinica (HKAS), Chinese Academy of Sciences, Kunming, China. Herbarium specimen for *S. rosigena* was also deposited in MFLU while its living culture in Mae Fah Luang University Culture Collection (MFLUCC). Facesoffungi and MycoBank numbers are provided as described in Jayasiri et al. (2015) and MycoBank (http://www.MycoBank.org) respectively. Species concepts are discussed following Jeewon and Hyde (2016).

### DNA extraction, PCR amplification and sequencing

Fresh mycelium from the culture of *S. rosigena* (MFLUCC 18-0387) scraped from the margin of colonies on MEA plates (incubated at room temperature for 4 weeks), and conidiomata of the new taxon (MFLU 18-0131) from natural substrate were used for DNA extraction. Around 20 conidiomata of the new taxon (MFLU 18-0131) were carefully picked from the sterilized material using a fine sterile needle, observed through a stereomicroscope and collected in a 1.5 ml micro-centrifuge tube for subsequent DNA extraction. Genomic DNA was extracted using Forensic DNA Kit (D3591-01, OMEGA bio-tek), following the manufacturer’s instructions. The loci LSU, ITS, *β*-*tub* and *rpb2* were amplified using primers LR0R/LR5 (Vilgalys and Hester 1990; Rehner and Samuels 1994), ITS5/ITS4 (White et al. 1990; Ward and Adams 1998), BT-2a/BT-2b (Glass and Donaldson 1995) and fRPB2-5F/fRPB2-7cR (Liu et al. 1999; Sung et al. 2007) respectively. Polymerase Chain Reactions (PCR) were conducted in an Applied Biosystems C1000 Touch^TM^ Thermal Cycler with the following PCR conditions for LSU, ITS, *β*-*tub* and *rpb2* regions: initial denaturation at 95 °C for 3 min followed by 34 cycles of denaturation at 95 °C for 30 s and 30 s of annealing and elongation at 72 °C for 1 min, and a final extension at 72 °C for 10 min. The annealing temperatures were 52 °C for LSU and 58 °C for ITS, *β*-*tub* and *rpb2*. The PCR reaction mixture, 25 µL in final volume, was composed of 0.3 µL of TaKaRa Ex-Taq DNA polymerase (TaKaRa, China), 2.5 µL of 10x Ex-Taq buffer (TaKaRa, China), 3.0 µL (2.5 μM) of dNTPs (TaKaRa, China), 1 µL of genomic DNA, 1 µL (0.4 μM) of each primer, and 16.2 µL of double-distilled H_2_O. Sequencing of PCR products was carried out with the same primers as mentioned above at the Beijing Biomed Gene Technology Co., Ltd, and Sangon Biotech, Shanghai China. The newly generated sequences were deposited in GenBank (Table 1).

### Phylogenetic analyses

Newly generated sequences from LSU, ITS, *β*-*tub* and *rpb2* during this study (Table 1) were analyzed with other sequences obtained from GenBank along with recently published relevant phylogenies (Wanasinghe et al. 2018; Liu et al. 2019). Sequences for each locus (LSU, ITS, *β-tub* and *rpb2*) were aligned using MAFFT V.7.036 (http://mafft.cbrc.jp/alignment/server/; Katoh et al. 2019), with L-INS-i Iterative refinement methods and manually improved when necessary in BioEdit v. 7.0 (Hall 2004). Phylogenetic analyses of the aligned data were based on maximum likelihood (ML) and Bayesian inference (BI) analyses with details as outlined by Tang et al. (2007, 2009).

**Table 1.** Taxa used in the phylogenetic analyses and corresponding GenBank accession numbers.

Taxa	Strain number	GenBank accession numbers			
		LSU	ITS	*β-tub*	*rpb2*
*Discosia artocreas*	CBS 124848^T^	MH554213	MH553994	MH554662	MH554903
Discosia aff. brasiliensis	NRBC 104198	AB593706	AB594774	N/A	N/A
*Discosia brasiliensis*	MFLUCC 12-0429 = NTCL094-2	KF827436	KF827432	KF827469	KF827473
	MFLUCC 12-0431 = NTCL095	KF827437	KF827433	KF827470	KF827474
	MFLUCC 12-0435 = NTCL097-2	KF827438	KF827434	KF827471	KF827475
*Discosia fagi*	MFLU 14-0299A =IT-722A^T^	KM678048	KM678040	N/A	N/A
	MFLU14-0299B = IT-722B	KM678047	KM678039	N/A	N/A
*Discosia italica*	MFLU 14-0298A = IT-712A^T^	KM678045	KM678042	N/A	N/A
	MFLU 14-0298B = IT-712B	KM678046	KM678043	N/A	N/A
	MFLU14-0298C = IT-712C	KM678044	KM678041	N/A	N/A
*Discosia macrozamiae*	CPC 32109	MH327856	MH327820	MH327895	N/A
*Discosia neofraxinea*	MFLUCC 12-0670 = NTIT469	KF827439	KF827435	KF827472	KF827476
	MFLU 15-0375^T^	KR072672	KR072673	N/A	N/A
*Discosia pini*	MAFF 410149	AB593708	AB594776	AB594174	N/A
Discosia aff. pleurochaeta	KT2192 = MAFF 242782	AB593714	AB594782	AB594180	N/A
	KT2179 = MAFF 242778	AB593709	AB594777	AB594175	N/A
	KT2188 = MAFF 242779	AB593713	AB594781	AB594179	N/A
*Discosia pseudoartocreas*	CBS 136438^T^	KF777214	KF777161	MH554672	MH554913
*Discosia querci*	MFLUCC 16-0642^T^	MG815830	MG815829	N/A	N/A
***Discosia ravennica***	**MFLU 18-0131^T^**	**MT376617**	**MT376615**	**MT393594**	**MW468059**
*Discosia rubi*	CBS 143893^T^	MH554334	MH554131	MH554804	MH555038
*Discosia tricellularis*	MAFF 237478	AB593730	AB594798	AB594189	N/A
*Discosia tricellularis*	NBRC 32705^T^	AB593728	AB594796	AB594188	N/A
*Discosia yakushimensis*	MAFF 242774 = NBRC 104194^T^	AB594796	AB594789	AB594187	N/A
*Pestalotiopsis hollandica*	CBS 265.33^T^	AB594188	KM199328	KM199388	MH554936
*Pseudopestalotiopsis cocos*	CBS 272.29^T^		NR_145246	KM199467	MH554938
*Sporocadus biseptatus*	CBS 110324 = MYC 754^T^	MH554179	MH553956	MH554615	MH554853
*Sporocadus cornicola*	CBS 143889 = CPC 23235	MH554326	MH554121	MH554794	MH555029
	MFLUCC 14-0448^T^	N/A	KU974967	N/A	N/A
*Sporocadus cotini*	CBS 139966 = MFLUCC 14-0623^T^	MH554222	MH554003	MH554675	MH554916
*Sporocadus incanus*	CBS 123003^T^	MH554210	MH553991	MH554659	MH554900
*Sporocadus lichenicola*	CBS 354.90 = NBRC 32677	MH554252	MH554035	MH554711	MH554948
	CPC 24528	MH554332	MH554127	MH554800	MH555036
	NBRC 32625 = IMI 079706^T^	MH883646	MH883643	MH883645	MH883647
*Sporocadus mali*	CBS 446.70^T^	MH554261	MH554049	MH554725	MH554960
*Sporocadus microcyclus*	CBS 424.95^T^	MH554258	MH554045	MH554721	MH554956
	CBS 887.68 = NBRC 32680	MH554280	MH554068	MH554744	MH554981
*Sporocadus multiseptatus*	CBS 143899 = CPC 26606^T^	MH554343	MH554141	MH554814	MH555047
*Sporocadus rosarum*	CBS 113832 = UPSC 2172	MH554189	MH553970	MH554629	MH554864
*Sporocadus rosigena*	CBS 116498	MH554200	MH553983	MH554642	MH554883
	CBS 129166 = MSCL 860	MH554215	MH553996	MH554665	MH554905
	CBS 182.50	MH554233	MH554013	MH554689	MH554926
	CBS 250.49	MH554245	MH554023	MH554699	MH554934
	CBS 466.96	MH554265	MH554052	MH554728	MH554965
	MFLU 16-0239^T^	MG829069	MG828958	N/A	N/A
***Sporocadus rosigena***	**MFLUCC 18-0387**	**MT376616**	**MT376614**	**MT393595**	**N/A**
*Sporocadus rotundatus*	CBS 616.83^T^	MH554273	MH554060	MH554737	MH554974
*Sporocadus sorbi*	MFLUCC 14-0469^T^	KT281911	KT284774	N/A	N/A
	CBS 160.25	MH554229	MH554008	MH554684	MH554924
*Sporocadus* sp.	CBS 506.71	MH554268	MH554055	MH554731	MH554968
*Sporocadus trimorphus*	CBS 114203 = UPSC 2430^T^	MH554196	MH553977	MH554636	MH554876

Abbreviations: **CBS**: Culture collection of the Westerdijk Fungal Biodiversity Institute, Utrecht, The Netherlands, **CPC**: Culture collection of Pedro Crous, housed at the Westerdijk Institute, **IMI**: International Mycological Institute, CABI-Bioscience, Egham, Bakeham Lane, United Kingdom, **MAFF**: Ministry of Agriculture, Forestry and Fisheries, Tsukuba, Ibaraki, Japan, **MFLU**: Mae Fah Luang University, Chiang Rai, Thailand, **MFLUCC**: Mae Fah Luang University Culture Collection, Chiang Rai, Thailand, **MSCL**: Microbial Strain Collection of Latvia, **NBRC**: Biological Resource Center, **UPSC**: Uppsala University Culture Collection of Fungi, Sweden. Types, ex-types and authentic strains are indicated with T. Newly generated sequences in this study are indicated in bold. “N/A” sequence is unavailable.

RAxML-HPC2 on XSEDE (v. 8.2.8) (Stamatakis et al. 2008; Stamatakis 2014) in the CIPRES Science Gateway platform (Miller et al. 2010) was used to generate the ML trees. Optimal ML tree search was conducted with 1000 separate runs, using the default algorithm of the program from a random starting tree for each run. The ultimate tree was selected among suboptimal trees from each run by comparing likelihood scores under the GTRGAMMA substitution model.

Bayesian analysis was executed in MrBayes v. 3. 1. 2 (Huelsenbeck and Ronquist 2001) through Markov Chain Monte Carlo (MCMC) sampling to calculate the posterior probabilities (PP) (Rannala and Yang 1996; Zhaxybayeva and Gogarten 2002). Partitioning of data was initially done by locus and then the parameters of the nucleotide substitution models for every partition were selected independently using MrModeltest v. 2.3 (Nylander 2004). Six Markov chains were run in parallel for 5M generations with trees being sampled every 1000^th^ generation. The distribution of log-likelihood scores was examined to determine the stationary phase for each search and to decide whether additional runs were required to reach convergence, using the program Tracer 1.5 (Rambaut and Drummond 2007). Convergence was declared when the average standard deviation of split frequencies at the end of the total MCMC generations was at 0.01. First 20% of generated trees was discarded as burn-in and the remaining 80% was used to calculate PP of the majority rule consensus tree (Dissanayake et al. 2020). The resulting trees were viewed in FigTree v. 1.4.0 (Rambaut 2012) and annotated in Microsoft PowerPoint (2013). The final alignment was registered in TreeBASE under the submission ID: 27601.

## Results

### Phylogenetic analyses

**Figure 1.**
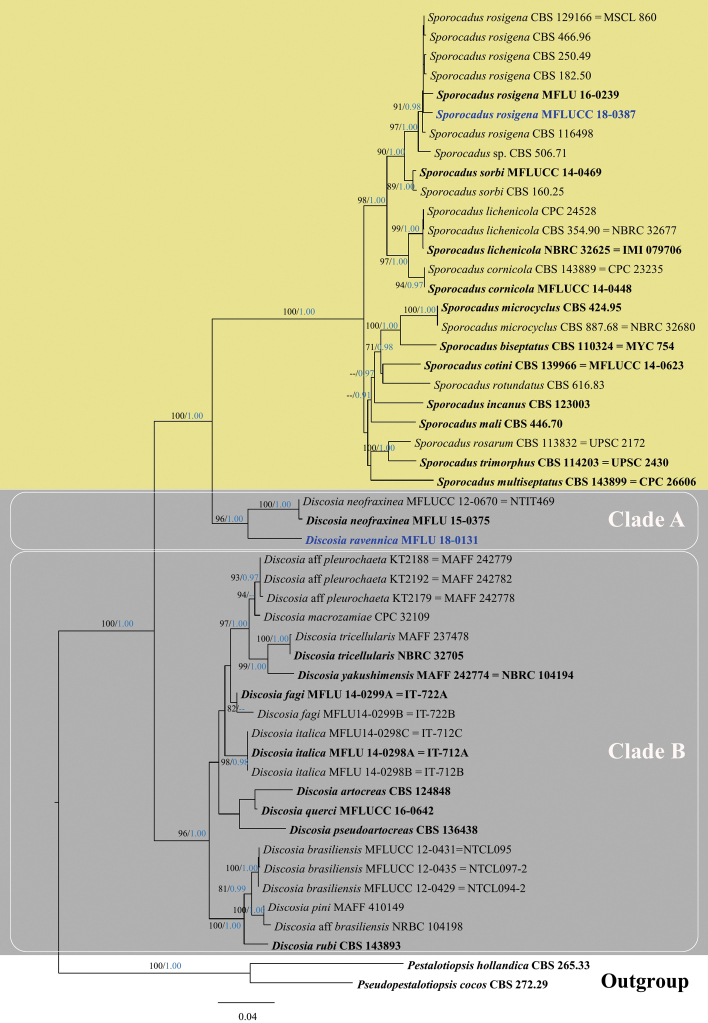
Phylogram generated from maximum likelihood (RAxML) based on analysis of a combined dataset of LSU, ITS, *β-tub* and *rpb2* sequence data. Bootstrap support values for ML equal to or greater than 70% (black) and Bayesian posterior probabilities (PP) equal to or greater than 0.90 (blue) are defined as ML/PP above or below the nodes. Type collections are in bold while the newly generated sequences are in blue bold type. The tree is rooted to *Pestalotiopsis hollandica* (CBS 265.33) and *Pseudopestalotiopsis cocos* (CBS 272.29). The scale bar represents the expected number of nucleotide substitutions per site.

The combined gene dataset (LSU, ITS, *β-tub* and *rpb2*) used to generate ML tree in Fig. 1 comprised 51 taxa including the newly generated sequences. *Pestalotiopsis hollandica* (CBS 265.33) and *Pseudopestalotiopsis cocos* (CBS 272.29) were selected as outgroup. The ML tree topology was similar to the one of the BI consensus tree. The best scoring RAxML tree with final optimization had a likelihood value of -15179.071239. The matrix had 1020 distinct alignment patterns, with 24.74% of gaps and completely undetermined characters. Estimated base frequencies were as follows: A= 0.247496, C= 0.245307, G= 0.252993, T= 0.254204, with substitution rates AC= 1.621276, AG= 6.173475, AT= 1.526832, CG= 1.406021, CT= 9.022198, GT= 1.000000; gamma distribution shape parameter α= 0.158554 and Tree-length = 1.305620.

*Discosia* taxa were divided into two separate clades (A and B). Clade A, consisting of 3 strains of *Discosia*, grouped with and was sister to *Sporocadus* with strong statistical support (100% ML, 1.00 PP). Clade B, comprising 21 strains of *Discosia*, was basal to both *Sporocadus* and clade A with strong statistical support (100% ML, 1.00 PP). Our strain MFLU 18-0131 was positioned in clade A, basal to both strains of *D. neofraxinea* (MFLU 15-0375 and MFLUCC 12-0670 = NTIT469), forming an independent lineage with good statistical support (96% ML/ 1.00 PP).

All the *Sporocadus* species formed a monophyletic clade with strong statistical support (100% ML, 1.00 PP). The strain MFLUCC 18-0387 from this study clustered with the other existing *S. rosigena* strains with a bootstrap support of 91% ML and 0.98 PP (Fig. 1).

### Taxonomy

#### Discosia ravennica

Taxon classificationFungiAmphisphaerialesDiscosiaceae

Bundhun, Jeewon, Camporesi, J.C. Kang & K.D. Hyde, sp. nov.

7170308F-6973-571D-AD49-8DD5F8C27217

837963

Facesoffungi Number: FoF07929

Figure 2

##### Etymology.

The specific epithet *ravennica* refers to the province of Ravenna, where the fungus was collected.

##### Holotype.

MFLU 18-0131

##### Description.

*Saprobic* on leaves of *Pyrus* sp. ***Sexual morph***: Undetermined. ***Asexual morph***: *Conidiomata* 45–70 µm high, 410–800 µm diam., stromatic, scattered to gregarious, superficial, rounded to unevenly outlined with complete margins, applanate, unilocular to bilocular, rugose, not glabrous, dull black, ostiolate. *Ostiole* 50–90 µm diam., circular to oval, opening to the exterior, central. *Conidiomatal wall* 10–20 µm thick at the base, dark brown in the outermost layer, comprising thick-walled cells of *textura angularis*, gradually becoming pale towards the inner layer; 10–20 µm thick near the apex, dark brown to black, made up of thick-walled cells of *textura epidermoidea*; interlocular wall composed of dark brown thick-walled cells of *textura prismatica*, becoming thin-walled and paler towards the outer layers. *Conidiophores* up to 40 µm high, originating from the innermost layer cells of the basal stroma, unbranched or at times branched, mostly 0–1-septate, rarely 2-septate or reduced to conidiogenous cells, cylindrical, hyaline, smooth. *Conidiogenous cells* 8–30 × 0.7–1.5 µm (x– = 14.3 × 1.1 µm, n = 15), subcylindrical to elongate-ampuliform, hyaline, smooth-walled, holoblastic. *Conidia* 12–16 × 1.5–3 µm (x– = 13.8 × 2.3 µm, n = 40) naviculate, to subcylindrical, narrow towards the base, straight or faintly curved, euseptate, mostly 3-septate, occasionally 2-septate, with septa thicker and darker than the periclinal wall, with cells unequal, hyaline to sub-hyaline, smooth-walled, without constriction at septa, bearing appendages on both apical and basal cells; basal cell 3–6 µm (x– = 3.8 µm) long, narrowly obconic, with truncate base bearing a conspicuous dehiscence scar; 2 median cells, together 6–10 µm (x– = 7.4 µm) long [second cell 4–6 µm (x– = 5.0 µm) long, close to apical cell, almost twice the size of the third cell 2–4 µm (x– = 3.0 µm) long, close to basal cell]; apical cell 3–5 µm (x– = 3.6 µm) long, subconical with acute apex, hyaline at apex and sub-hyaline below; appendages tubular, faintly broad at the base, unbranched, flexuous; appendage on apical cell 5–17 µm (x– = 10.1 µm) long, single, polar; appendage on basal cell 4–17 µm (x– = 9.4 µm) long, single, inserted slightly above conidium base.

**Figure 2.**
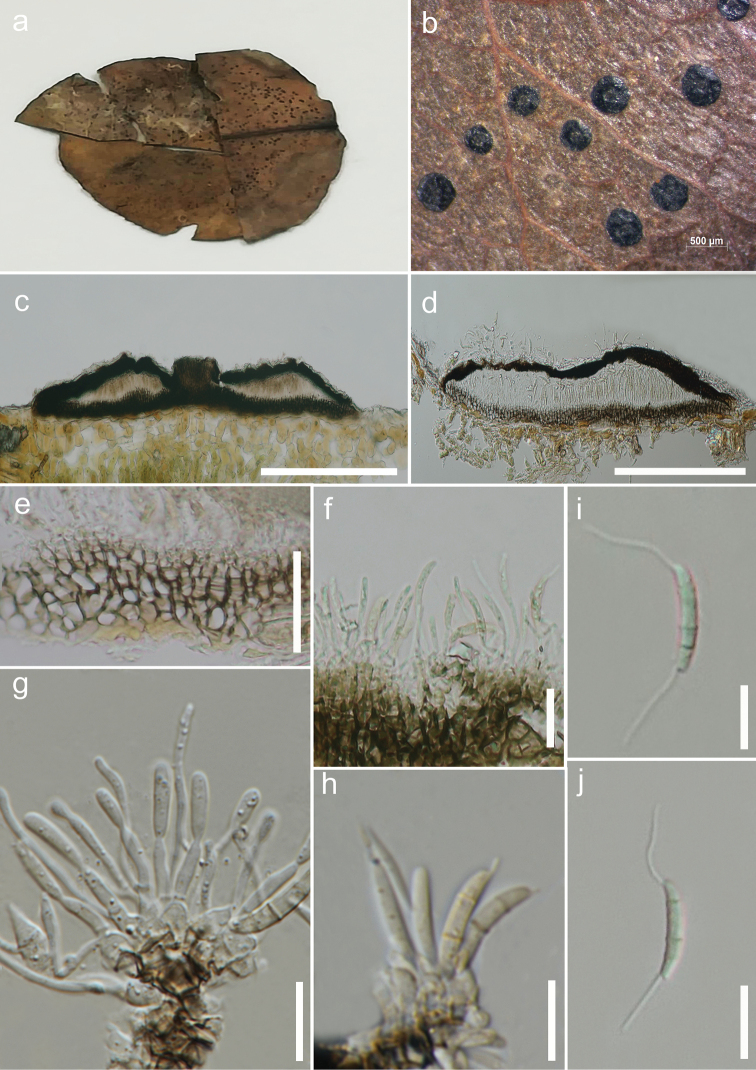
*Discosia ravennica* (MFLU 18-0131, holotype) **a** Herbarium specimen **b** Conidiomata on the host **c, d** Vertical sections of conidiomata **e** Conidioma wall at the base **f–h** Conidiogenous cells and developing conidia **i, j** Conidia. Scale bars: 500 μm (**b**); 200 μm (**c, d**); 10 μm (**e, g–j**); 20 μm (**f**).

##### Material examined.

ITALY. Province of Ravenna [RA], Oriolo dei Fichi– Faenza; on dead land leaves of *Pyrus* sp.; 24 Dec. 2017; Erio Camporesi; IT 3632 (MFLU 18-0131, ***holotype***; HKAS 104973, isotype).

##### Notes.

In the present study, no culture could be obtained for *D. ravennica* despite several trials on various media including MEA, potato dextrose agar, corn meal agar or water agar at different incubation conditions, the reason for which the species was subjected to direct DNA extraction from conidiomata. *Discosia ravennica* is morphologically similar to *D. neofraxinea* in terms of superficial conidiomata, which are not glabrous and 3-septate conidia with cells of unequal length. It also closely resembles *D. fraxinea* (Schwein.) Nag Raj (1993) in having uni-to bi-locular applanate conidiomata and naviculate to subcylindrical 3-septate conidia with cells of unequal length. The new species, however, also differs from the latter two species as mentioned in Table 2.

#### Sporocadus rosigena

Taxon classificationFungiAmphisphaerialesDiscosiaceae

F. Liu, L. Cai & Crous, in Liu, Bonthond, Groenewald, Cai & Crous, Stud. Mycol. 92: 402 (2018)

211989ED-0B3B-5D38-A063-B97E2EAF4545

Facesoffungi number: FoF07930

Figure 3

≡ *Seimatosporium rosicola* Wanas., Goonas., Camporesi, & K.D. Hyde, in Wanasinghe et al., Fungal Diversity 193 (2018)

##### Description.

*Saprobic* on *Quercus ilex* L. ***Sexual morph***: Illustrated in Wanasinghe et al. (2018). ***Asexual morph***: *Conidiomata* (on host) 115–145 µm diam., 70–130 µm high, acervular, solitary to aggregated, semi-immersed, black; (on MEA) 50–70 µm diam., acervular, solitary to aggregated, erumpent, black. *Conidiophores* (on MEA) cylindrical, branched, hyaline, smooth, up to 30 µm long. *Conidiogenous cells* (on MEA) 7–18 × 2–3 µm (x– = 10.1 × 2.1 µm, n = 20) cylindrical, enteroblastic, annellidic, integrated or discrete, hyaline, determinate, smooth. *Conidia* (on MEA) 12–15 × (3–) 5–7 µm (x– = 13.5 × 5.4 µm, n = 47), obovoid, ellipsoid, broad fusiform or subcylindrical, straight or curved, hyaline when immature, pale to moderate brown at maturity, with 3 transverse, thick, darker septa, rarely constricted at the septa, often obtuse at both ends, or well rounded, smooth-walled, no appendage or sheath; basal cell obconic with a truncate base, pale brown or hyaline, thin-walled, 1–2.5 µm long (x– = 2 µm); two median cells doliiform, hyaline or pale brown, turning brown at maturity, together 5–7 µm long (x– = 6.1 µm), second cell from the base 1–3 µm long (x– = 2.5 µm), third cell from the base 1.4–4 µm long (x– = 2.6 µm); apical cell conical with obtuse or rounded apex, concolorous with the median cells, 1.8–3.5 µm long (x– = 2.5 µm).

**Figure 3.**
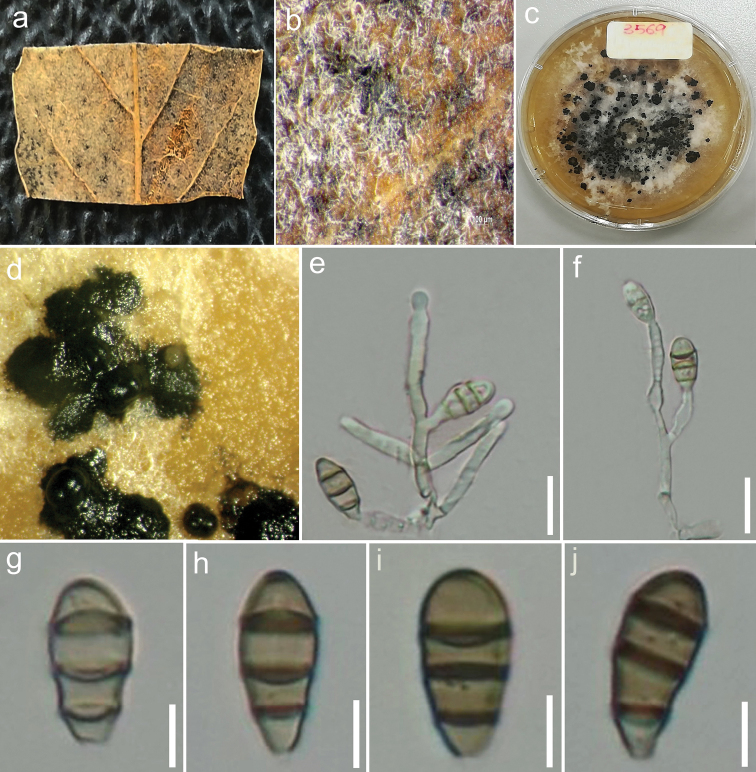
*Sporocadus rosigena* (MFLU 17-2803) **a** Leaf of *Quercus ilex* L **b** Close-up of conidiomata on host **c** Upper view of colony on MEA**d** Conidiomata in culture (MFLUCC 18-0387) **e, f** Different stages of conidiogenesis (MFLUCC 18-0387) **g–j** Conidia (MFLUCC 18-0387). Scale bars: 10 μm (**e, f**); 5 μm (**g–j**).

##### Culture characteristics.

Colonies on MEA reaching 2–3 cm diam. after 11 days at 18 °C in darkness, filamentous, circular, flat with entire margin, white from above, reverse pale yellow.

##### Material examined.

ITALY. Province of Forlì-Cesena, Fiumana di Predappio; on dead land leaf of *Quercus ilex* L. (Fagaceae); 20 Nov. 2017; Erio Camporesi; IT 3569 (MFLU 17-2803); living culture MFLUCC 18-0387.

##### Notes.

*Sporocadus rosigena* from the present study shares similar morphology with the other *S. rosigena* strains in having almost obovoid, ellipsoid or fusiform to subcylindrical conidia (Wanasinghe et al. 2018; Liu et al. 2019). Pairwise comparison of DNA sequence data of the isolate MFLUCC 18-0387 with the other strains of *S. rosigena* revealed very minor differences and thus, the strain MFLUCC 18-0387 is considered as *S. rosigena*.

## Discussion

*Discosia ravennica* sp. nov. forms an independent lineage, basal to the two strains of *D. neofraxinea* (96% ML/ 1.00 PP) (Fig. 1). It is different from *D. neofraxinea* in its unilocular to bilocular, applanate conidiomata along with elongate-ampulliform conidiogenous cells and conidia smaller in size (Table 2). With regard to DNA sequence data comparison, *D. ravennica* differs from both strains of *D. neofraxinea* (MFLU 15-0375 and MFLUCC 12-0670 = NTIT469) in having 14 out of 531 (2.6 %) and 8 out of 512 (1.6%) different base pairs (bp) in the ITS alignments respectively. Moreover, 13 bp out of 229 (5.7%) and 82 bp out of 832 (9.9%) differences in the *ß-tub* and *rpb2* alignments respectively can be observed between *D. ravennica* and *D. neofraxinea* (MFLUCC 12-0670 = NTIT469). Sequence data of *ß-tub* and *rpb2* are not available for the strain of *D. neofraxinea* (MFLU 15-0375) in GenBank and hence could not be compared. Similarly, no molecular data for *D. fraxinea* are accessible in GenBank, following which the new species, *D. ravennica*, has been delineated based on morphology (Table 2). The 5.7% and 9.9% differences in nucleotides in *ß-tub* and *rpb2* respectively may acceptably support the establishment of a new species (Jeewon and Hyde 2016). Following this assumption along with the above-mentioned morphological differences and high statistical support, *D. ravennica* is herein established as a new species.

**Table 2.** Features distinguishing *Discosia ravennica*, *D. fraxinea* and *D. neofraxinea*.

Features	*Discosia ravennica* (this study)	*Discosia fraxinea* (Nag Raj 1993)	*Discosia neofraxinea* (Senanayake et al. 2015)
Host occurrence	Leaves of *Pyrus* sp.	*Amelanchier vulgaris, Crataegus* sp., *Fraxinus americana*, *Populus* sp., *Sorbus americana* and undetermined leaves	Leaves of *Fagus sylvatica*
Known distribution	Italy	Austria, France, Germany, U.S.A.	Italy
Conidiomata	Superficial	Erumpent	Superficial
Basal stroma	Composed of cells of *textura angularis*	Composed of cells of *textura prismatica*	Composed of cells of *textura prismatica*
Conidiogenous cells	8–30 × 0.7–1.5 µm Subcylindrical to elongate-ampuliform	7–40 × 1.5–2.5 µm Subcylindrical to langeniform or ampuliform	6–40 × 1–2 μm Cylindrical
Conidia	12–16 × 1.5–3 µm (x– = 13.8 × 2.3 µm)	12.5–19 × 2.5–3.5 µm (x– = 16.2 × 3 µm)	15–18 × 2.5–3.5 μm (x– = 16 × 3 μm)

A peculiar finding from our DNA sequence analyses is the placement of *D. neofraxinea* and *D. ravennica*. Both of them constitute a strongly supported independent clade (clade A) basal to species of *Sporocadus*. One might argue that given their distinct phylogenetic nature, a new genus accommodating these two species might be a possibility. However, in this particular scenario, we would rather take a more conservative and lumping taxonomic approach and maintain the latter two species in *Discosia*. The reasons we would advocate are that there is a lot of morphological resemblance between members of clades A and B. For instance, when we compare *D. neofraxinea* and *D. ravennica* (clade A) with the type species, *D. artocreas* (clade B), they all have stromatic conidiomata, conidiophores which arise from the upper cell layer of the basal stroma, and hyaline to sub-hyaline, usually 3-septate conidia bearing two appendages (Nag Raj 1993; Senanayake et al. 2015; Liu et al. 2019). The main difference is that *D. neofraxinea* and *D. ravennica* have the third cell of their conidia from the base longer than the second cell while *D. artocreas* has the second cell of its conidia from the base longer than the third cell (Nag Raj 1993) or both median cells of almost equal length (Liu et al. 2019). However, this distinctive characteristic is not sufficient enough for the establishment of a new genus. It might be that the genus is paraphyletic, but until more species are recovered and analyzed to provide further taxonomic insights, we refrain from making any taxonomic amendments. It might also be possible that there is a need to establish species complexes given the wide intraspecies variation as we have seen in other genera such as *Phyllosticta* (Norphanphoun et al. 2020).

The second recovered species from this study, *Sporocadus rosigena*, clusters with other *S. rosigena* strains in a well-supported clade (91% ML / 0.98 PP) in our 4-gene phylogeny (Fig. 1). The latter shows similar topology to the 5-gene phylogeny reported by Liu et al. (2019). *Sporocadus rosigena* has earlier been reported as saprobic or endophytic on species of *Rosa*, *Rubus*, *Pyrus* (Rosaceae), *Rhododendron* (Ericaceae) and *Vitis* (Vitaceae) (Wanasinghe et al. 2018; Liu et al. 2019). In this study, the species was found from *Quercus ilex* (Fagaceae) and is therefore introduced as a new host record. Different fungi have equally been reported from *Quercus ilex* in Italy; for instance, the genera *Alternaria* (Lunghini et al. 2013), *Beltrania* (Pirozynski 1963), *Endothia* (Spaulding 1961), *Monochaetia* (Nag Raj 1993), *Neognomoniopsis* (Crous et al. 2019), *Pestalotia* (Nag Raj 1993), *Xylaria* and *Zygosporium* (Lunghini et al. 2013), indicating a broad diversity of fungi on the same host. All *Sporocadus* species in their asexual stage possess 3-septate, obovoid, fusoid to cylindrical conidia, which do not have any appendage. The only exceptions are *S. trimorphus* and *S. rosarum*, which are known to produce conidia both with and without appendages (Liu et al. 2019).

Fungal diversity and classification are always ever-changing and require an ongoing assessment (Hyde and Soytong 2008; Jeewon et al. 2017). This becomes especially essential in cases where taxa are described from genera which usually accommodate pathogens. *Discosia*, for instance, is known to comprise the plant pathogen *D. yakushimensis* which causes leaf spots on plants such as *Symplocos prunifolia* (Tanaka et al. 2011). Identifying novel species in a genus may also potentially imply the discovery of emerging pathogens which can cause damage to crops of economic importance (Jayawardena et al. 2019a, 2019b). Evolutionary relationships and ecological roles of fungi have been reported to be intricately linked to the emergence of new species (Zhang et al. 2008; Hyde et al. 2020). However, such phenomena also extend to the recognition of existing species from new hosts, as is the case for *S. rosigena* in the present study. Documenting records from new hosts has become useful repertoires for mycologists who aim to understand evolution of fungi, host jumping, expanding host diversity and adaptations to different environmental conditions (Hyde et al. 2020). These are equally important for proper quarantine measures, whereby potential pathogens or species known to have a wide host diversity are to be closely monitored with a view to avoid unintentional disturbance to a specific environment (Cai et al. 2011).

## Supplementary Material

XML Treatment for Discosia ravennica

XML Treatment for Sporocadus rosigena
